# *catena*-Poly[copper(II)-{μ_3_-4,4′-dichloro-2,2′-[butane-1,4-diylbis(nitrilo­methanyl­yl­idene)]diphenolato-κ^4^ *N*,*O*:*N*′,*O*′:*O*′}]

**DOI:** 10.1107/S1600536811009974

**Published:** 2011-03-26

**Authors:** Hadi Kargar, Reza Kia

**Affiliations:** a Chemistry Department, Payame Noor University, Tehran 19395-4697, I. R. of Iran; b X-ray Crystallography Lab., Plasma Physics Research Center, Science and Research Branch, Islamic Azad University, Tehran, Iran

## Abstract

The asymmetric unit of the title coordination polymer, [Cu(C_18_H_16_Cl_2_N_2_O_2_)]_*n*_, consists of a Schiff base complex in which the Cu^II^ atom adopts a square-pyramidal coordination geometry, being coordinated by two N and two O atoms of symmetry-related ligands and by a third O atom from a complex related by an inversion center. In the structure, a crystallographic twofold rotation axis bis­ects the central C—C bonds of the *n*-butyl spacers of the designated Schiff base ligands, making symmetry-related dimeric units, which are twisted around Cu^II^ atoms in a bis-bidentate coordination mode. In the crystal, these dimeric units are connected through the third bridging Cu—O bonds [2.3951 (13) Å], forming one-dimensional coordination polymers, which propagate along [001]. Furthermore, inter­molecular π–π inter­actions [centroid–centroid distance = 3.811 (1) Å] stabilize the crystal packing.

## Related literature

For van der Waals radii, see: Bondi (1964 ▶). For background to coordination polymers, see: Kido & Okamoto (2002 ▶); Li *et al.* (2006 ▶); Eddaoudi *et al.* (2001 ▶); Dietzel *et al.* (2005 ▶). For background to bis-bidentate Schiff base complexes, see: Hannon *et al.* (1999 ▶); Lavalette *et al.* (2003 ▶). For the synthesis and structural variations of Schiff base complexes, see: Granovski *et al.* (1993 ▶); Elmali *et al.* (2000 ▶).
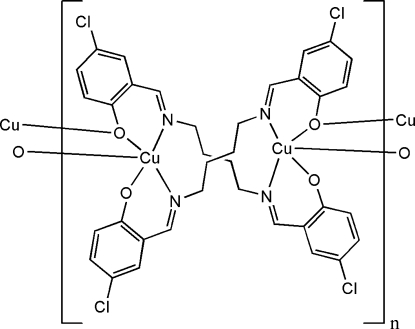


## Experimental

### 

#### Crystal data

[Cu(C_18_H_16_Cl_2_N_2_O_2_)]
*M* *_r_* = 426.77
Monoclinic, 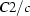

*a* = 23.7249 (5) Å
*b* = 10.5067 (2) Å
*c* = 15.2460 (3) Å
β = 116.988 (1)°
*V* = 3386.52 (12) Å^3^
*Z* = 8
Mo *K*α radiation
μ = 1.62 mm^−1^
*T* = 100 K
0.42 × 0.23 × 0.17 mm

#### Data collection

Bruker SMART APEXII CCD area-detector diffractometer
Absorption correction: multi-scan (*SADABS*; Bruker, 2001 ▶) *T*_min_ = 0.547, *T*_max_ = 0.768
30759 measured reflections
7465 independent reflections
5511 reflections with *I* > 2σ(*I*)
*R*_int_ = 0.048

#### Refinement

*R*[*F* ^2^ > 2σ(*F* ^2^)] = 0.041
*wR*(*F* ^2^) = 0.108
*S* = 1.03
7465 reflections
226 parameters
H-atom parameters constrained
Δρ_max_ = 1.07 e Å^−3^
Δρ_min_ = −0.70 e Å^−3^

### 

Data collection: *APEX2* (Bruker, 2007 ▶); cell refinement: *SAINT* (Bruker, 2007 ▶); data reduction: *SAINT*; program(s) used to solve structure: *SHELXS97* (Sheldrick, 2008 ▶); program(s) used to refine structure: *SHELXL97* (Sheldrick, 2008 ▶); molecular graphics: *SHELXTL* (Sheldrick, 2008 ▶); software used to prepare material for publication: *SHELXTL* and *PLATON* (Spek, 2009 ▶).

## Supplementary Material

Crystal structure: contains datablocks global, I. DOI: 10.1107/S1600536811009974/su2262sup1.cif

Structure factors: contains datablocks I. DOI: 10.1107/S1600536811009974/su2262Isup2.hkl

Additional supplementary materials:  crystallographic information; 3D view; checkCIF report

## Figures and Tables

**Table 1:** Hydrogen-bond geometry (Å, °)

*D*—H⋯*A*	*D*—H	H⋯*A*	*D*⋯*A*	*D*—H⋯*A*
C18—H18*A*⋯O1	0.97	2.28	2.973 (2)	127

**Fig. 1.**
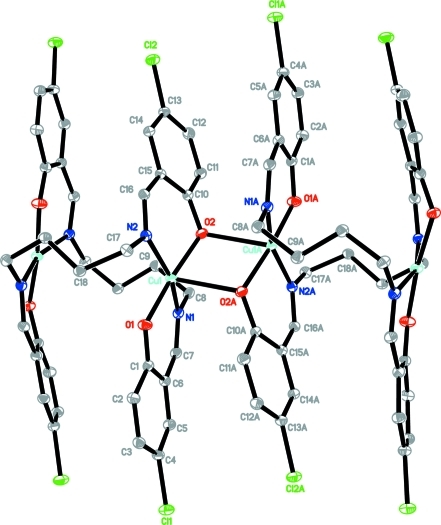
The molecular structure of the title complex, showing 40% probability displacement ellipsoids and the atomic numbering [H-atoms have been omitted for clarity; symmetry code: (A) -x, -y+1, -z].

**Fig. 2.**
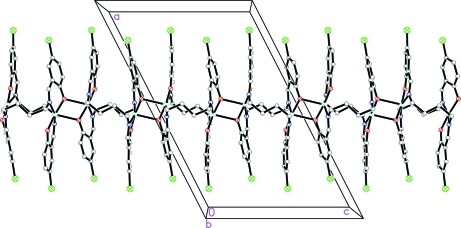
The crystal packing, viewed down the b-axis, of the title complex, showing the one-dimensional coordination chain propagating along the c-axis [H-atoms have been omitted for clarity].

## supplementary crystallographic information

### Comment

The design and construction of metal-organic coordination polymers (MOCPs) have attracted considerable attention, not only for their novel topologies but also for their potential in the area of magnetic applications and functional materials (Kido & Okamoto, 2002; Li *et al.*, 2006; Eddaoudi *et al.*, 2001; Dietzel *et al.*, 2005). One of the key strategies in the construction of metal-organic coordination polymers is to select suitable bi- or multi-dentate bridging ligands. Among these, bis-bidentate *NN*- or *NO*-donor Schiff base ligands with aliphatic and aromatic spacers (Hannon *et al.*, 1999; Lavalette *et al.*, 2003) have attracted much attention because of the flexibility in their coordination modes and the resulting intermolecular interactions. The long chain aliphatic spacers or rigid aromatic spacers with large bite angles in these ligands favour the bis-bidentate coordination mode and allow the ligands to accomodate metal centers in one unit of the ligand. On the other hand, Schiff bases are one of the most prevalent ligands in coordination chemistry and their complexes are some of the most important stereochemical models in transition metal-organic chemistry, with their ease of preparation and structural variations (Granovski *et al.*, 1993; Elmali *et al.*, 2000).

The molecular structure of the title complex (Fig. 1) consists of symmetry-related dimers in which the Schiff base ligands are twisted around Cu^II ^centers in a bis-bidentate coordination mode, having a crystallographic twofold rotation axis which passes through the central C—C bonds of the *n*-butyl spacers [C9—C9A^i^ and C18—C18A^i^; symmetry code: (i) -*x*, *y*, -*z* + 1/2 ].

In the crystal the dimer units are connected through Cu—O bonds, forming one-diensional coordination polymer running along the *c* axis (Fig. 2), in which the Cu^II^ atom adopts a square-based pyramidal coordination geometry. The Cu^II^ atoms are supported by the two nitrogen and oxygen atoms of the symmetry-related ligands and a third oxygen atom of neighboring complexes. The lengths of the intermolecular Cu1—O2^i^ bonds [2.3951 (13) Å; symetry code (i) -*x*, -*y*+1, -*z*] is significantly shorter than the sum of the van der Waals (vdW) radii of these atoms [Cu, 1.43Å and O, 1.52 Å; Bondi, 1964]. There are different non-bonded internuclear Cu···Cu distances. The longer one is separated by the butyl spacers [4.672 Å], and the shorter one is in the centrosymmetric Cu_2_O_2_ rectangular unit [3.299 Å]. Furthermore, intermolecular π-π interactions stabilize the crystal packings with centroid to centroid distances of 3.811 (1)Å [*Cg*1 and *Cg*2 are the centroids of the rings (C1–C6) and (C10–C15)]. There are also C—H···O interactions present (Table 1).

### Experimental

The title complex was synthesized by the template method of mixing an ethanolic solution (50 ml) of 5-chlorosalicylaldeyde (4 mmol), 1,4-butanediamine (2 mmol), and CuCl_2_.4H_2_O (2.1 mmol). After stirring at reflux conditions for 2 h, the solution was filtered and the resulting green solid was crystallized from ethanol, giving single crystals suitable for X-ray diffraction. Spectoscopic and analytical data are given in the archived CIF.

### Refinement

All H atoms were positioned geometrically and constrained to refine with the parents atoms using the riding-model approximation, with C—H = 0.93 - 0.97Å and *U*_iso_(H) = 1.2 *U*_eq_(C).

### Crystal data

**Table:**

[Cu(C_18_H_16_Cl_2_N_2_O_2_)]	*F*(000) = 1736
*M**_r_* = 426.77	*D*_x_ = 1.674 Mg m^−^^3^
Monoclinic, *C*2/*c*	Mo *K*α radiation, λ = 0.71073 Å
Hall symbol: -C 2yc	Cell parameters from 7283 reflections
*a* = 23.7249 (5) Å	θ = 2.4–34.8°
*b* = 10.5067 (2) Å	µ = 1.62 mm^−^^1^
*c* = 15.2460 (3) Å	*T* = 100 K
β = 116.988 (1)°	Block, green
*V* = 3386.52 (12) Å^3^	0.42 × 0.23 × 0.17 mm
*Z* = 8	

### Data collection

**Table:**

Bruker SMART APEXII CCD area-detector diffractometer	7465 independent reflections
Radiation source: fine-focus sealed tube	5511 reflections with *I* > 2σ(*I*)
graphite	*R*_int_ = 0.048
φ and ω scans	θ_max_ = 35.2°, θ_min_ = 2.4°
Absorption correction: multi-scan (*SADABS*; Bruker, 2001)	*h* = −38→38
*T*_min_ = 0.547, *T*_max_ = 0.768	*k* = −15→16
30759 measured reflections	*l* = −24→23

### Refinement

**Table:**

Refinement on *F*^2^	Primary atom site location: structure-invariant direct methods
Least-squares matrix: full	Secondary atom site location: difference Fourier map
*R*[*F*^2^ > 2σ(*F*^2^)] = 0.041	Hydrogen site location: inferred from neighbouring sites
*wR*(*F*^2^) = 0.108	H-atom parameters constrained
*S* = 1.03	*w* = 1/[σ^2^(*F*_o_^2^) + (0.0534*P*)^2^ + 2.3966*P*] where *P* = (*F*_o_^2^ + 2*F*_c_^2^)/3
7465 reflections	(Δ/σ)_max_ = 0.001
226 parameters	Δρ_max_ = 1.07 e Å^−^^3^
0 restraints	Δρ_min_ = −0.69 e Å^−^^3^

### Special details

**Table:**

Experimental. Spectoscopic and analytical data:FTIR (KBr, cm^-1^): νmax 1622 (versus), 1533 (s), 1465 (s), 1386 (s), 1317 (s), 1195 (m), 1176 (m), 821 (s), 705 (s). Anal. Calc. for C_18_H_16_Cl_2_CuN_2_O_2_: 50.66; H, 3.78; N, 6.56 %. Found: C, 50.70; H, 3.66; N, 6.57 %.
Geometry. All e.s.d.'s (except the e.s.d. in the dihedral angle between two l.s. planes) are estimated using the full covariance matrix. The cell e.s.d.'s are taken into account individually in the estimation of e.s.d.'s in distances, angles and torsion angles; correlations between e.s.d.'s in cell parameters are only used when they are defined by crystal symmetry. An approximate (isotropic) treatment of cell e.s.d.'s is used for estimating e.s.d.'s involving l.s. planes.
Refinement. Refinement of *F*^2^ against ALL reflections. The weighted *R*-factor *wR* and goodness of fit *S* are based on *F*^2^, conventional *R*-factors *R* are based on *F*, with *F* set to zero for negative *F*^2^. The threshold expression of *F*^2^ > σ(*F*^2^) is used only for calculating *R*-factors(gt) *etc*. and is not relevant to the choice of reflections for refinement. *R*-factors based on *F*^2^ are statistically about twice as large as those based on *F*, and *R*- factors based on ALL data will be even larger.

### Fractional atomic coordinates and isotropic or equivalent isotropic displacement parameters (Å^2^)

**Table:**

	*x*	*y*	*z*	*U*_iso_*/*U*_eq_	
Cu1	0.022690 (9)	0.53850 (2)	0.116076 (15)	0.01452 (6)	
Cl1	0.362651 (19)	0.48963 (5)	0.38428 (4)	0.02231 (9)	
Cl2	−0.31688 (2)	0.50558 (5)	−0.08067 (4)	0.02457 (10)	
O1	0.09481 (6)	0.63334 (13)	0.20125 (10)	0.0197 (2)	
O2	−0.04854 (6)	0.44838 (12)	0.01836 (9)	0.0169 (2)	
N1	0.06650 (7)	0.37212 (14)	0.17172 (10)	0.0158 (3)	
N2	−0.03106 (6)	0.69745 (15)	0.09189 (10)	0.0151 (3)	
C1	0.15334 (8)	0.59527 (17)	0.24600 (12)	0.0160 (3)	
C2	0.20162 (8)	0.68856 (17)	0.28841 (13)	0.0177 (3)	
H2A	0.1904	0.7735	0.2877	0.021*	
C3	0.26477 (8)	0.65589 (18)	0.33059 (13)	0.0178 (3)	
H3A	0.2956	0.7187	0.3563	0.021*	
C4	0.28228 (8)	0.52825 (17)	0.33464 (13)	0.0170 (3)	
C5	0.23719 (8)	0.43461 (18)	0.30031 (13)	0.0173 (3)	
H5A	0.2495	0.3497	0.3064	0.021*	
C6	0.17232 (8)	0.46601 (17)	0.25576 (12)	0.0157 (3)	
C7	0.12726 (8)	0.36225 (17)	0.22349 (12)	0.0169 (3)	
H7A	0.1435	0.2806	0.2417	0.020*	
C8	0.02982 (8)	0.25245 (18)	0.14935 (13)	0.0175 (3)	
H8A	0.0587	0.1812	0.1749	0.021*	
H8B	0.0064	0.2424	0.0785	0.021*	
C9	−0.01643 (8)	0.25036 (18)	0.19396 (12)	0.0176 (3)	
H9A	−0.0438	0.3242	0.1709	0.021*	
H9B	−0.0428	0.1751	0.1709	0.021*	
C10	−0.10753 (8)	0.46309 (17)	0.00258 (12)	0.0150 (3)	
C11	−0.14972 (8)	0.35895 (18)	−0.03223 (13)	0.0189 (3)	
H11A	−0.1347	0.2802	−0.0402	0.023*	
C12	−0.21273 (8)	0.37172 (19)	−0.05460 (13)	0.0197 (3)	
H12A	−0.2396	0.3018	−0.0766	0.024*	
C13	−0.23591 (8)	0.48954 (19)	−0.04415 (13)	0.0180 (3)	
C14	−0.19608 (8)	0.59274 (18)	−0.00739 (12)	0.0170 (3)	
H14A	−0.2119	0.6704	0.0009	0.020*	
C15	−0.13129 (7)	0.58023 (17)	0.01757 (12)	0.0151 (3)	
C16	−0.09196 (8)	0.69309 (17)	0.05320 (12)	0.0155 (3)	
H16A	−0.1128	0.7699	0.0475	0.019*	
C17	−0.00209 (8)	0.82531 (17)	0.12060 (12)	0.0167 (3)	
H17A	−0.0338	0.8894	0.0852	0.020*	
H17B	0.0315	0.8335	0.1015	0.020*	
C18	0.02494 (8)	0.85016 (17)	0.23129 (12)	0.0161 (3)	
H18A	0.0563	0.7854	0.2665	0.019*	
H18B	0.0463	0.9319	0.2462	0.019*	

### Atomic displacement parameters (Å^2^)

**Table:**

	*U*^11^	*U*^22^	*U*^33^	*U*^12^	*U*^13^	*U*^23^
Cu1	0.01204 (9)	0.01378 (10)	0.01660 (10)	0.00038 (7)	0.00551 (7)	−0.00146 (7)
Cl1	0.01324 (16)	0.0221 (2)	0.0287 (2)	0.00137 (15)	0.00701 (15)	0.00028 (16)
Cl2	0.01322 (17)	0.0314 (3)	0.0279 (2)	−0.00119 (16)	0.00826 (16)	−0.00369 (18)
O1	0.0139 (5)	0.0149 (6)	0.0256 (6)	0.0014 (4)	0.0048 (5)	−0.0040 (5)
O2	0.0132 (5)	0.0174 (6)	0.0205 (5)	−0.0002 (4)	0.0079 (4)	−0.0032 (4)
N1	0.0160 (6)	0.0149 (7)	0.0179 (6)	0.0002 (5)	0.0089 (5)	−0.0007 (5)
N2	0.0155 (6)	0.0150 (7)	0.0148 (6)	−0.0003 (5)	0.0068 (5)	−0.0005 (5)
C1	0.0149 (7)	0.0162 (8)	0.0169 (7)	0.0012 (6)	0.0074 (6)	−0.0015 (6)
C2	0.0167 (7)	0.0139 (8)	0.0205 (7)	0.0006 (6)	0.0066 (6)	−0.0014 (6)
C3	0.0165 (7)	0.0171 (8)	0.0184 (7)	−0.0019 (6)	0.0066 (6)	−0.0016 (6)
C4	0.0133 (6)	0.0179 (8)	0.0180 (7)	0.0010 (6)	0.0055 (6)	−0.0005 (6)
C5	0.0158 (7)	0.0158 (8)	0.0197 (7)	0.0014 (6)	0.0075 (6)	0.0011 (6)
C6	0.0161 (7)	0.0148 (8)	0.0168 (7)	−0.0003 (6)	0.0079 (6)	−0.0002 (6)
C7	0.0165 (7)	0.0159 (8)	0.0187 (7)	0.0018 (6)	0.0084 (6)	−0.0001 (6)
C8	0.0173 (7)	0.0164 (8)	0.0213 (7)	−0.0021 (6)	0.0108 (6)	−0.0024 (6)
C9	0.0170 (7)	0.0177 (8)	0.0191 (7)	−0.0019 (6)	0.0090 (6)	−0.0008 (6)
C10	0.0144 (6)	0.0158 (8)	0.0150 (6)	0.0008 (5)	0.0067 (5)	−0.0004 (6)
C11	0.0165 (7)	0.0176 (8)	0.0218 (8)	−0.0010 (6)	0.0080 (6)	−0.0034 (6)
C12	0.0176 (7)	0.0208 (9)	0.0205 (7)	−0.0044 (6)	0.0085 (6)	−0.0032 (6)
C13	0.0133 (7)	0.0234 (9)	0.0171 (7)	−0.0011 (6)	0.0066 (6)	−0.0007 (6)
C14	0.0142 (7)	0.0190 (8)	0.0172 (7)	0.0028 (6)	0.0065 (6)	0.0000 (6)
C15	0.0137 (6)	0.0161 (8)	0.0146 (6)	−0.0004 (5)	0.0057 (5)	−0.0007 (6)
C16	0.0147 (7)	0.0150 (8)	0.0154 (7)	0.0019 (5)	0.0057 (5)	−0.0005 (5)
C17	0.0177 (7)	0.0142 (8)	0.0174 (7)	−0.0012 (6)	0.0075 (6)	0.0008 (6)
C18	0.0143 (7)	0.0151 (8)	0.0178 (7)	−0.0013 (6)	0.0063 (5)	−0.0012 (6)

### Geometric parameters (Å, °)

**Table:**

Cu1—O1	1.8948 (13)	C7—H7A	0.9300
Cu1—O2	1.9201 (12)	C8—C9	1.531 (2)
Cu1—N1	2.0139 (15)	C8—H8A	0.9700
Cu1—N2	2.0308 (15)	C8—H8B	0.9700
Cu1—O2^i^	2.3951 (13)	C9—C9^ii^	1.523 (3)
Cl1—C4	1.7503 (17)	C9—H9A	0.9700
Cl2—C13	1.7488 (17)	C9—H9B	0.9700
O1—C1	1.301 (2)	C10—C11	1.414 (2)
O2—C10	1.3172 (19)	C10—C15	1.415 (2)
O2—Cu1^i^	2.3951 (13)	C11—C12	1.381 (2)
N1—C7	1.296 (2)	C11—H11A	0.9300
N1—C8	1.478 (2)	C12—C13	1.393 (3)
N2—C16	1.290 (2)	C12—H12A	0.9300
N2—C17	1.482 (2)	C13—C14	1.379 (3)
C1—C6	1.417 (3)	C14—C15	1.412 (2)
C1—C2	1.421 (2)	C14—H14A	0.9300
C2—C3	1.379 (2)	C15—C16	1.453 (2)
C2—H2A	0.9300	C16—H16A	0.9300
C3—C4	1.397 (3)	C17—C18	1.531 (2)
C3—H3A	0.9300	C17—H17A	0.9700
C4—C5	1.370 (2)	C17—H17B	0.9700
C5—C6	1.411 (2)	C18—C18^ii^	1.529 (3)
C5—H5A	0.9300	C18—H18A	0.9700
C6—C7	1.448 (2)	C18—H18B	0.9700
			
O1—Cu1—O2	173.80 (6)	C9—C8—H8A	109.2
O1—Cu1—N1	92.00 (6)	N1—C8—H8B	109.2
O2—Cu1—N1	90.18 (6)	C9—C8—H8B	109.2
O1—Cu1—N2	89.39 (6)	H8A—C8—H8B	107.9
O2—Cu1—N2	90.32 (6)	C9^ii^—C9—C8	113.17 (17)
N1—Cu1—N2	162.14 (6)	C9^ii^—C9—H9A	108.9
O1—Cu1—O2^i^	93.09 (5)	C8—C9—H9A	108.9
O2—Cu1—O2^i^	80.87 (5)	C9^ii^—C9—H9B	108.9
N1—Cu1—O2^i^	97.28 (5)	C8—C9—H9B	108.9
N2—Cu1—O2^i^	100.42 (5)	H9A—C9—H9B	107.8
C1—O1—Cu1	127.92 (12)	O2—C10—C11	119.37 (15)
C10—O2—Cu1	124.88 (11)	O2—C10—C15	122.73 (15)
C10—O2—Cu1^i^	120.03 (10)	C11—C10—C15	117.89 (15)
Cu1—O2—Cu1^i^	99.13 (5)	C12—C11—C10	121.35 (17)
C7—N1—C8	116.63 (15)	C12—C11—H11A	119.3
C7—N1—Cu1	123.02 (13)	C10—C11—H11A	119.3
C8—N1—Cu1	120.25 (11)	C11—C12—C13	119.84 (17)
C16—N2—C17	116.13 (15)	C11—C12—H12A	120.1
C16—N2—Cu1	122.32 (12)	C13—C12—H12A	120.1
C17—N2—Cu1	121.53 (10)	C14—C13—C12	120.80 (15)
O1—C1—C6	124.21 (16)	C14—C13—Cl2	120.31 (14)
O1—C1—C2	118.33 (16)	C12—C13—Cl2	118.88 (14)
C6—C1—C2	117.46 (15)	C13—C14—C15	119.85 (16)
C3—C2—C1	121.43 (17)	C13—C14—H14A	120.1
C3—C2—H2A	119.3	C15—C14—H14A	120.1
C1—C2—H2A	119.3	C14—C15—C10	120.14 (16)
C2—C3—C4	119.86 (16)	C14—C15—C16	117.42 (16)
C2—C3—H3A	120.1	C10—C15—C16	122.34 (14)
C4—C3—H3A	120.1	N2—C16—C15	126.57 (16)
C5—C4—C3	120.49 (16)	N2—C16—H16A	116.7
C5—C4—Cl1	120.47 (14)	C15—C16—H16A	116.7
C3—C4—Cl1	119.04 (13)	N2—C17—C18	112.71 (14)
C4—C5—C6	120.53 (17)	N2—C17—H17A	109.0
C4—C5—H5A	119.7	C18—C17—H17A	109.0
C6—C5—H5A	119.7	N2—C17—H17B	109.0
C5—C6—C1	120.00 (16)	C18—C17—H17B	109.0
C5—C6—C7	117.61 (16)	H17A—C17—H17B	107.8
C1—C6—C7	122.37 (15)	C18^ii^—C18—C17	113.78 (17)
N1—C7—C6	126.34 (17)	C18^ii^—C18—H18A	108.8
N1—C7—H7A	116.8	C17—C18—H18A	108.8
C6—C7—H7A	116.8	C18^ii^—C18—H18B	108.8
N1—C8—C9	112.04 (14)	C17—C18—H18B	108.8
N1—C8—H8A	109.2	H18A—C18—H18B	107.7
			
N1—Cu1—O1—C1	21.62 (15)	O1—C1—C6—C5	176.08 (16)
N2—Cu1—O1—C1	−176.19 (15)	C2—C1—C6—C5	−4.2 (2)
O2^i^—Cu1—O1—C1	−75.79 (15)	O1—C1—C6—C7	−5.4 (3)
N1—Cu1—O2—C10	125.77 (14)	C2—C1—C6—C7	174.30 (16)
N2—Cu1—O2—C10	−36.38 (14)	C8—N1—C7—C6	−178.71 (15)
O2^i^—Cu1—O2—C10	−136.88 (16)	Cu1—N1—C7—C6	4.9 (2)
N1—Cu1—O2—Cu1^i^	−97.35 (5)	C5—C6—C7—N1	−171.74 (16)
N2—Cu1—O2—Cu1^i^	100.50 (5)	C1—C6—C7—N1	9.7 (3)
O2^i^—Cu1—O2—Cu1^i^	0.0	C7—N1—C8—C9	117.57 (17)
O1—Cu1—N1—C7	−16.53 (14)	Cu1—N1—C8—C9	−65.94 (17)
O2—Cu1—N1—C7	157.66 (14)	N1—C8—C9—C9^ii^	−65.42 (14)
N2—Cu1—N1—C7	−110.7 (2)	Cu1—O2—C10—C11	−149.56 (13)
O2^i^—Cu1—N1—C7	76.84 (14)	Cu1^i^—O2—C10—C11	81.66 (18)
O1—Cu1—N1—C8	167.21 (12)	Cu1—O2—C10—C15	31.4 (2)
O2—Cu1—N1—C8	−18.60 (12)	Cu1^i^—O2—C10—C15	−97.34 (16)
N2—Cu1—N1—C8	73.0 (2)	O2—C10—C11—C12	−176.62 (16)
O2^i^—Cu1—N1—C8	−99.42 (12)	C15—C10—C11—C12	2.4 (3)
O1—Cu1—N2—C16	−164.27 (14)	C10—C11—C12—C13	0.8 (3)
O2—Cu1—N2—C16	21.92 (14)	C11—C12—C13—C14	−3.0 (3)
N1—Cu1—N2—C16	−69.7 (2)	C11—C12—C13—Cl2	175.83 (14)
O2^i^—Cu1—N2—C16	102.69 (13)	C12—C13—C14—C15	1.7 (3)
O1—Cu1—N2—C17	13.94 (12)	Cl2—C13—C14—C15	−177.05 (13)
O2—Cu1—N2—C17	−159.87 (12)	C13—C14—C15—C10	1.6 (2)
N1—Cu1—N2—C17	108.6 (2)	C13—C14—C15—C16	178.22 (16)
O2^i^—Cu1—N2—C17	−79.09 (12)	O2—C10—C15—C14	175.38 (15)
Cu1—O1—C1—C6	−14.3 (2)	C11—C10—C15—C14	−3.6 (2)
Cu1—O1—C1—C2	166.02 (12)	O2—C10—C15—C16	−1.0 (3)
O1—C1—C2—C3	−175.20 (16)	C11—C10—C15—C16	179.95 (16)
C6—C1—C2—C3	5.1 (2)	C17—N2—C16—C15	178.78 (15)
C1—C2—C3—C4	−1.8 (3)	Cu1—N2—C16—C15	−2.9 (2)
C2—C3—C4—C5	−2.4 (3)	C14—C15—C16—N2	169.93 (16)
C2—C3—C4—Cl1	177.60 (14)	C10—C15—C16—N2	−13.6 (3)
C3—C4—C5—C6	3.3 (3)	C16—N2—C17—C18	101.27 (17)
Cl1—C4—C5—C6	−176.79 (13)	Cu1—N2—C17—C18	−77.05 (16)
C4—C5—C6—C1	0.2 (3)	N2—C17—C18—C18^ii^	−63.30 (14)
C4—C5—C6—C7	−178.41 (16)		

Symmetry codes: (i) −*x*, −*y*+1, −*z*; (ii) −*x*, *y*, −*z*+1/2.

### Hydrogen-bond geometry (Å, °)

**Table:**

*D*—H···*A*	*D*—H	H···*A*	*D*···*A*	*D*—H···*A*
C18—H18A···O1	0.97	2.28	2.973 (2)	127
